# Diagnostic Accuracy of Hand-Held Fundus Camera and Artificial Intelligence in Diabetic Retinopathy Screening

**DOI:** 10.3390/biomedicines12010034

**Published:** 2023-12-22

**Authors:** Martina Tomić, Romano Vrabec, Đurđica Hendelja, Vilma Kolarić, Tomislav Bulum, Dario Rahelić

**Affiliations:** 1 Department of Ophthalmology, Vuk Vrhovac University Clinic for Diabetes, Endocrinology and Metabolic Diseases, Merkur University Hospital, Dugi dol 4a, 10000 Zagreb, Croatia; 2 Department of Diabetes and Endocrinology, Vuk Vrhovac University Clinic for Diabetes, Endocrinology and Metabolic Diseases, Merkur University Hospital, Dugi dol 4a, 10000 Zagreb, Croatia; 3 School of Medicine, University of Zagreb, Šalata 3, 10000 Zagreb, Croatia; 4 School of Medicine, Catholic University of Croatia, Ilica 242, 10000 Zagreb, Croatia; 5 School of Medicine, Josip Juraj Strossmayer University, Josipa Huttlera 4, 31000 Osijek, Croatia

**Keywords:** diabetic retinopathy, screening, slit-lamp fundoscopy, fundus camera, artificial intelligence

## Abstract

Our study aimed to assess the role of a hand-held fundus camera and artificial intelligence (AI)-based grading system in diabetic retinopathy (DR) screening and determine its diagnostic accuracy in detecting DR compared with clinical examination and a standard fundus camera. This cross-sectional instrument validation study, as a part of the International Diabetes Federation (IDF) Diabetic Retinopathy Screening Project, included 160 patients (320 eyes) with type 2 diabetes (T2DM). After the standard indirect slit-lamp fundoscopy, each patient first underwent fundus photography with a standard 45° camera VISUCAM Zeiss and then with a hand-held camera TANG (Shanghai Zhi Tang Health Technology Co., Ltd.). Two retina specialists independently graded the images taken with the standard camera, while the images taken with the hand-held camera were graded using the DeepDR system and an independent IDF ophthalmologist. The three screening methods did not differ in detecting moderate/severe nonproliferative and proliferative DR. The area under the curve, sensitivity, specificity, positive predictive value, negative predictive value, positive likelihood ratio, negative likelihood ratio, kappa (ĸ) agreement, diagnostic odds ratio, and diagnostic effectiveness for a hand-held camera compared to clinical examination were 0.921, 89.1%, 100%, 100%, 91.4%, infinity, 0.11, 0.86, 936.48, and 94.9%, while compared to the standard fundus camera were 0.883, 83.2%, 100%, 100%, 87.3%, infinity, 0.17, 0.78, 574.6, and 92.2%. The results of our study suggest that fundus photography with a hand-held camera and AI-based grading system is a short, simple, and accurate method for the screening and early detection of DR, comparable to clinical examination and fundus photography with a standard camera.

## 1. Introduction

Diabetes is one of the fastest-growing global health emergencies of the 21st century that has reached alarming levels and is projected to affect 783 million people by 2045 [1]. Diabetes is also a significant cause of disability and is still the leading cause of preventable blindness in the adult working population, nontraumatic amputations, and renal failure. Despite the growing awareness of diabetes, its complications continue to represent a significant public health problem with high health expenditure [1]. Diabetic retinopathy (DR) represents a crucial complication of diabetes leading to vision loss and, finally, blindness in the adult working population [2]. About 22% of patients with diabetes have DR, but over 10% have vision-threatening DR (VTDR), including proliferative DR (PDR) and diabetic macular edema (DME) [3]. By 2045, the number of people with DR and VTDR is projected to increase from 103.12 million and 47.37 million in 2020 to 160.50 million and 73.43 million, respectively.

In clinical settings, it is essential to diagnose DR in its early asymptomatic stages and to start on time with adequate therapy, preventing vision loss and blindness. Screening for DR is the best and most cost-effective method to avoid blindness [4,5]. However, screening every person with diabetes for DR with standard ophthalmological examinations is not optimal considering the limited number of ophthalmologists, and represents an inefficient use of resources. The minimum examinations for the screening of DR include a vision examination (before pupil dilation) and a retinal examination adequate for DR classification [6,7]. Several low-cost fundus cameras are widely available, and fundus photography telemedicine has become a good option for DR screening [8,9,10]. Fundus cameras can be handled by educated nurses or trained photographers taking high-quality retinal images that can further be reviewed for interpretation and DR grading by an ophthalmologist or an entirely artificial intelligence (AI)-based automated system [11]. In real-time, the trained system decides whether a person requires a referral to an ophthalmologist, and it is much cheaper than having ophthalmologists screen each person with diabetes. The first such AI-based automated system for DR identification was approved in April 2018 by the US Food and Drug Administration (FDA) [12], and since then, many other systems have been developed and introduced [13,14,15].

Recently, fuzzy-based image edge detection algorithms for blood vessel detection in retinal images have also become available [16]. The performance and accuracy of vessel segmentation approaches are based on a combination of image preprocessing techniques, global thresholding, and image postprocessing techniques [17,18]. A framework based on hybrid 2D–3D convolutional neural networks is developed to obtain a continuous 3D automated surface segmentation of the retinal layer, which is essential and challenging in analyzing optical coherence tomography (OCT) [19]. To overcome the lack of precision of these methods preventing medical professionals from completely trusting the results generated, explainable AI has been introduced to help increase the interpretability of the methods [20]. A systematic review investigating the test accuracy of AI-based grading of fundus images in DR screening suggests that AI-based systems are more sensitive than human graders and could be safe to use in clinical practice, but have variable specificity [21].

**Figure 1:**
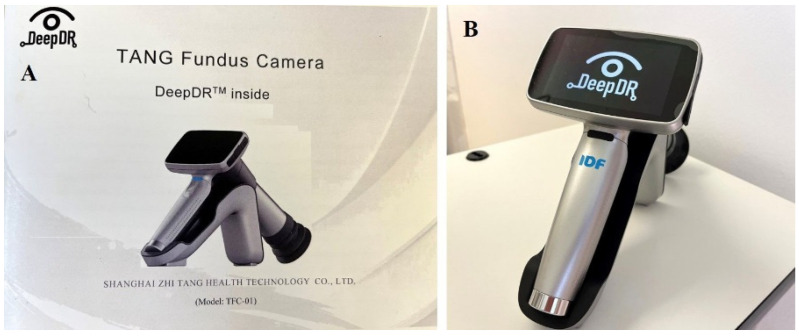
Instruction manual (**A**) and TANG hand-held fundus camera (**B**).

In July 2019, before the COVID-19 outbreak, the Republic of Croatia was, thanks to Professor Dario Rahelić, president of the Croatian Association for Diabetes and Metabolic Disorders and chair of the International Diabetes Federation (IDF) Young Leaders in Diabetes Programme, included in the IDF Diabetic Retinopathy Screening Project and for this purpose, received a hand-held fundus camera TANG, Shanghai Zhi Tang Health Technology Co., Ltd. (Shanghai China) (Fundoscope ID: EUR-04) (Figure 1) with DR screening software (DeepDR) that used the AI-based automated DR grading system for analyzing the retinal images captured by the camera. This IDF project and a partnership with the Fred Hollows Foundation highlighted the importance of integrating screening for DR ongoing care for people living with diabetes in developing countries worldwide and providing DR screening to all people with diabetes every one to two years [22]. The IDF also encouraged all people with diabetes who have not had their retinas screened in the past two years to speak to a health professional about receiving retinopathy screening as soon as possible.

As a part of the IDF Diabetic Retinopathy Screening Project, this study aimed to assess the role of a hand-held fundus camera and AI-based grading system in DR screening and determine its diagnostic accuracy in detecting DR compared with clinical examination and photography using the standard fundus camera.

## 2. Materials and Methods

### 2.1. Study Design and Patients

This cross-sectional instrument validation study, as a part of the IDF Diabetic Retinopathy Screening Project, was conducted in Vuk Vrhovac University Clinic for Diabetes, Endocrinology and Metabolic Diseases, Merkur University Hospital in Zagreb, Croatia, following the Declaration of Helsinki and approved by the Hospital’s Ethics Committee. All study participants received written and oral information about the study and signed the written informed consent.

A total of 160 patients (320 eyes) with type 2 diabetes (T2DM) consecutively referred to the ophthalmology department of the tertiary care specialist diabetes clinic over three months between September 2019 and December 2019 were randomly selected and included in the study by the first author, M.T., during her routine clinical work. At the inclusion visit, after signing the informed consent, the first author obtained a medical history regarding diabetes duration and other eye conditions and diseases. Patients with other posterior eye segment diseases (macular degeneration, the central retinal artery, vein, or branches occlusion) or anterior and posterior eye segment diseases that did not allow fundus visualization and photography (previous ocular trauma, acute infection, ocular surface diseases and irregularities, mature cataract, opacities, and vitreous hemorrhages) and patients with poor cooperation were not included in the study.

### 2.2. Ophthalmologic Retinal Examination and Fundus Photography

**Figure 2:**
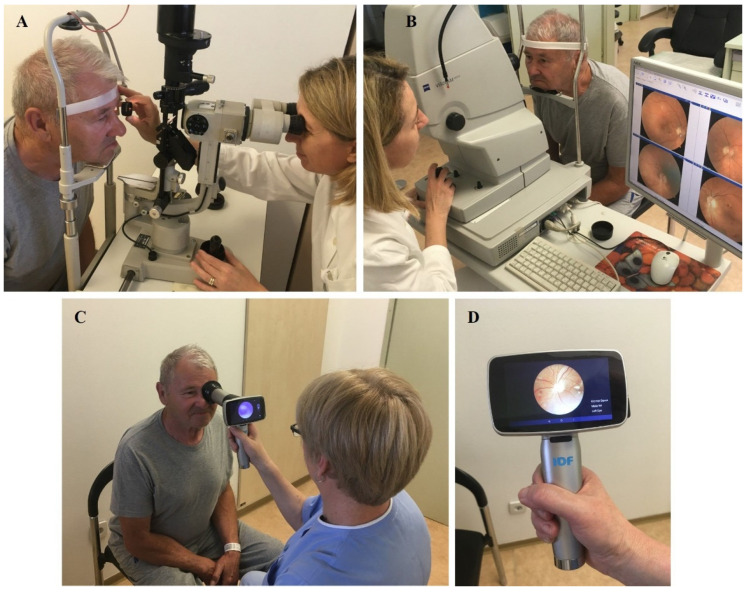
Standard clinical indirect slit-lamp fundus examination (**A**), photography with a standard (**B**) and a hand-held fundus camera (**C**,**D**).

After signing the informed consent and pupil dilation with eye drops containing 0.5% tropicamide, each patient first underwent a standard clinical examination—indirect slit-lamp fundoscopy (Figure 2A)—then fundus photography with a standard 45° fundus camera VISUCAM Zeiss (Carl Zeiss Meditec AG, Goeschwitzer Str. 51-52, 07745 Jena, Germany (Figure 2B), and finally fundus photography with a hand-held fundus camera TANG (Shanghai Zhi Tang Health Technology Co., Ltd.) (Shanghai China) (Figure 2C,D).

A biomicroscopic indirect slit-lamp fundus examination and color fundus photography of two fields (macula-centered, optic disc-centered) of both eyes with a standard VISUCAM Zeiss camera was performed by the first author, M.T. Two-field 45° photographs consisted of the first field image (macula-centered) covering the temporal area and optic disc and the second field image (optic disc-centered) covering the nasal area [23,24]. Two-field photography has the advantage of detecting DR in the nasal retina that single-field photography could otherwise miss. After that, the first, M.T., and the second author, R.V., two medical retina specialists, independently graded the photographs and assigned a DR grade using the proposed international clinical diabetic retinopathy and diabetic macular edema disease severity ratings [25]. Since there was no case where the experts assigned different grades, there was no need for a third grader.

Color fundus photography of two fields (macula-centered, optic disc-centered) of both eyes with a hand-held TANG camera was conducted, according to the IDF Diabetic Retinopathy Screening Project guidelines [26], by the ophthalmology nurse Đ.H. and graded using the AI-based automated software (DeepDR) and an independent IDF ophthalmologist from Brussels, Belgium. Fundus image reports from AI and the IDF ophthalmologist contained an image quality assessment, diagnosis, and advice. If the images were of sufficient quality, there were two possible results: (1) diagnose: no DR or mild nonproliferative DR (NPDR), and advice: regular follow-up; (2) diagnose: moderate/severe NPDR or PDR, and advice: refer to an ophthalmologist.

### 2.3. Statistical Analysis

Statistical analysis was performed, and the graphs were created using the software package Statistica™ 14.0.1 (TIBCO Software Inc., Palo Alto, CA, USA) and SPSS 23.0. (IBM, Armonk, NY, USA). The Kolmogorov–Smirnov test was used to evaluate the normality of data distribution, and the Leven test was used to evaluate the homogeneity of variance. The results were expressed as numbers or percentages for categorical variables and the mean ± SD or median (min-max) for continuous variables. For continuous data, the differences between groups were tested using the Kruskal–Wallis and one-way ANOVA tests. Scheffe’s post-hoc test was used where needed. For categorical data testing, the Chi-square test was used. A receiver operating characteristic (ROC) curve, the area under the ROC curve (AUC), sensitivity and specificity, predictive values, likelihood ratios, kappa agreement, diagnostic odds ratio, and diagnostic effectiveness (accuracy) were used to assess the ability of the hand-held fundus camera TANG and AI-based grading system in DR screening and determine its diagnostic accuracy in detecting DR compared to clinical examination and photography using the standard fundus camera. To calculate all diagnostic accuracy measures, the examined eyes were divided into two groups: eyes with no retinopathy and those with any retinopathy (mild/moderate/severe NPDR/PDR). A *p*-value < 0.05 was considered statistically significant in all analyses.

## 3. Results

This study included 320 eyes of 160 T2DM (89 male/71 female) with a median age of 65 (45–83) years and a median diabetes duration of 14 (2–33) years. Their mean best-corrected visual acuity (BCVA) was 0.98 ± 0.10, and the mean intraocular pressure (IOP) was 15.21 ± 1.01 mmHg. Sixteen (5%) eyes had primary open-angle glaucoma and were regularly treated with local antiglaucoma medications. Thirty-six (11.25%) eyes had a clear crystalline lens, 230 (71.87%) had initial cataract, and 54 (16.88%) eyes were pseudophakic due to previous cataract surgery. 

Based on the photography with a hand-held fundus camera, 202 (63.1%) eyes in this study had no DR, 58 (18.1%) had mild or moderate NPDR, and 60 (18.8%) eyes had severe NPDR or PDR. Their basic characteristics are presented in Table 1. The groups did not significantly differ in gender (Chi-square test, *p* = 0.058), age, and diabetes duration (Kruskal–Wallis test, *p* = 0.408, *p* = 0.161) nor in the mean IOP (one-way ANOVA test, *p* = 0.776), the prevalence of glaucoma, and the status of intraocular lens (Chi-square test, *p* = 0.612, *p* = 0.447). The only difference among the examined eyes was observed in BCVA (one-way ANOVA test, *p* = 0.027). The eyes with no retinopathy had significantly better BCVA than those with severe NPDR or PDR (Scheffe test, *p* = 0.029). 

**Figure 3:**
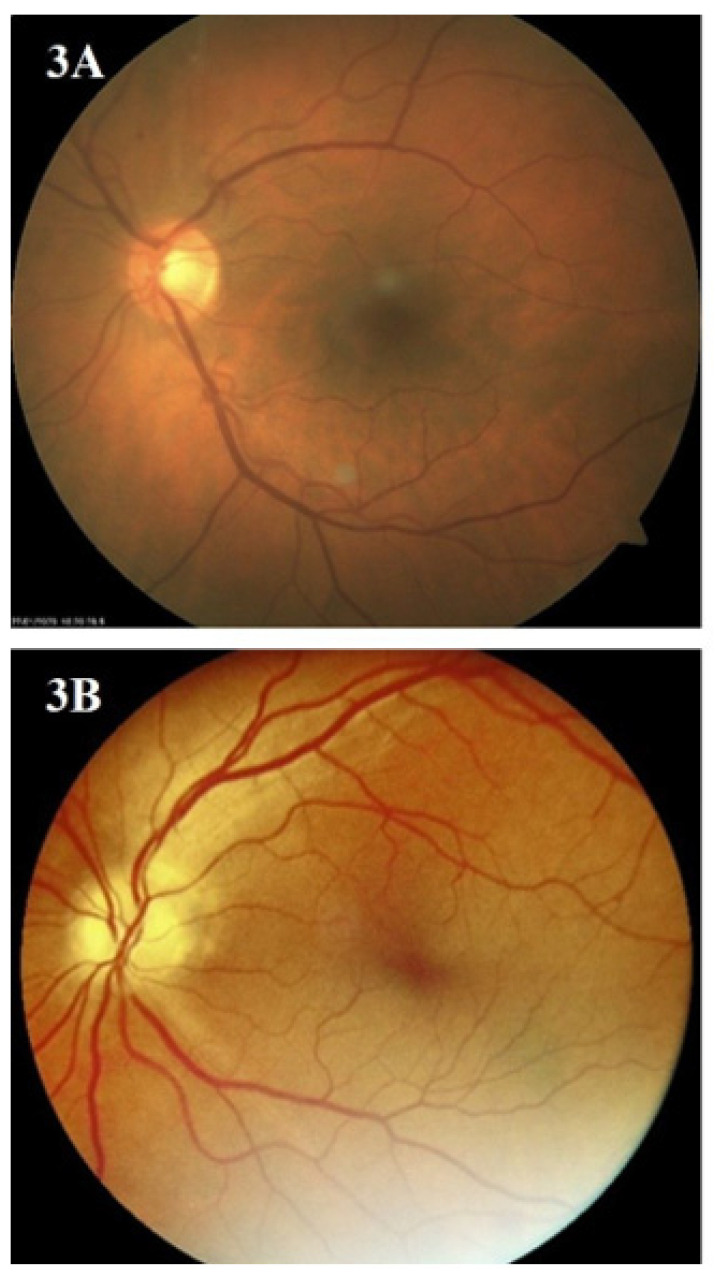
Images of the same patients taken by standard (**A**) and hand-held fundus camera (**B**).

**Figure 4:**
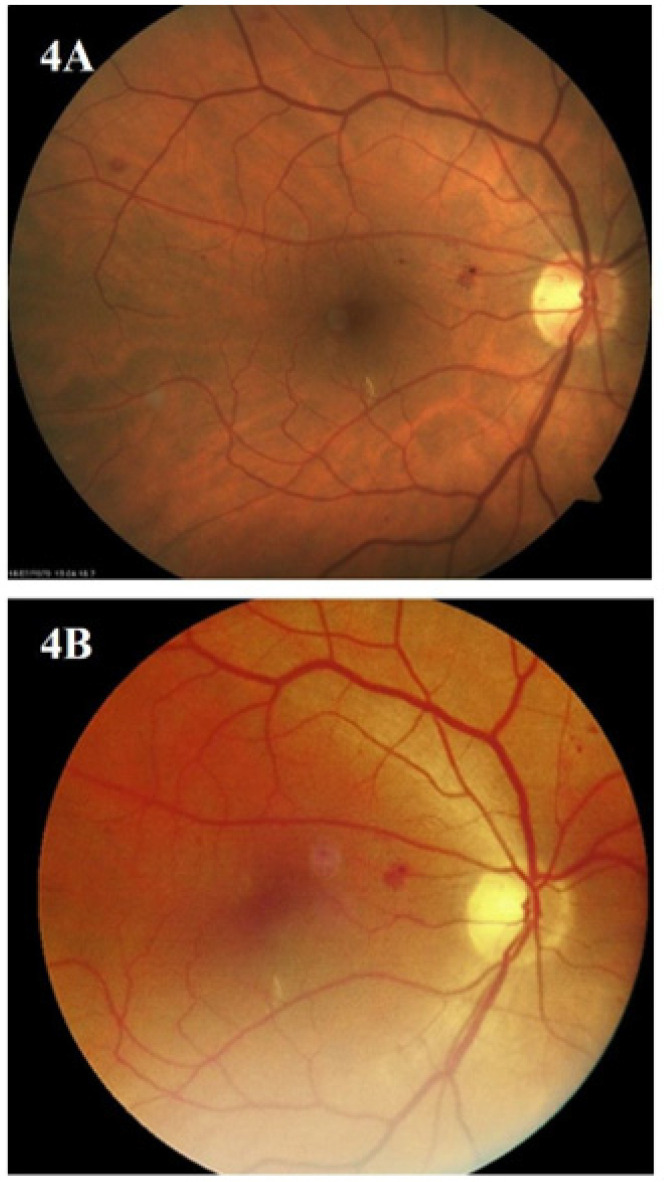
Images of the same patients taken by standard (**A**) and hand-held fundus camera (**B**).

**Figure 5:**
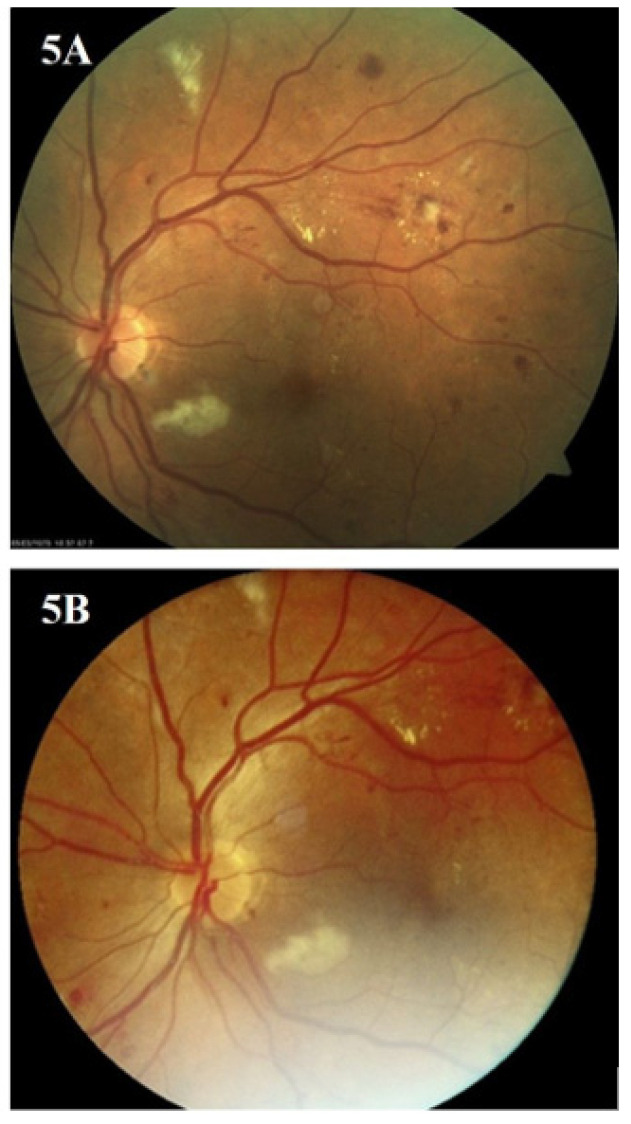
Images of the same patients taken by standard (**A**) and hand-held fundus camera (**B**).

After evaluation by AI and an IDF ophthalmologist, the images of 248 (77.5%) eyes taken by the hand-held camera were rated as good quality, while images of 72 (22.5%) eyes were of medium quality. Not a single image was considered low-quality or unreadable. Figure 3, Figure 4 and Figure 5 present the images of the same patients taken by the standard (A) and hand-held fundus camera (B).

**Figure 6:**
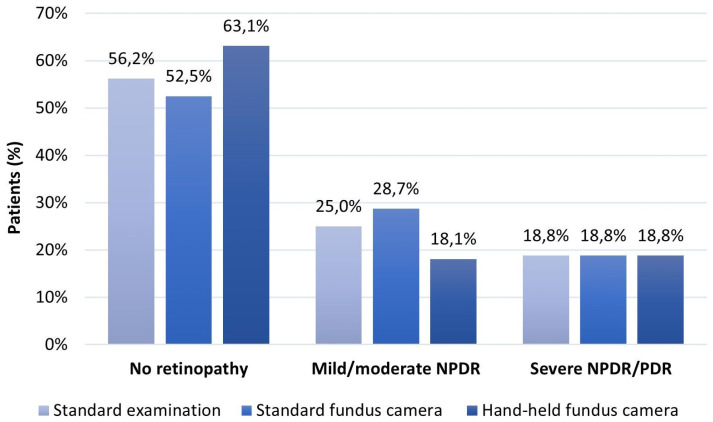
DR degree assessed by standard clinical examination, standard, and hand-held fundus camera.

When comparing the reports of the hand-held camera with the standard clinical examination and the standard camera in DR degree assessment, there were no differences in detecting severe NPDR and PDR between the three methods (Table 2, Figure 6). However, the most significant discrepancy in DR degree assessment was in the eyes with no DR and those with signs of mild NPDR (single microaneurysms). Out of the 202 eyes with no DR screened by the hand-held camera, the signs of mild NPDR were detected in 22 (6.9%) eyes screened by the standard clinical examination and in 34 (10.6%) eyes by the standard camera.

**Figure 7:**
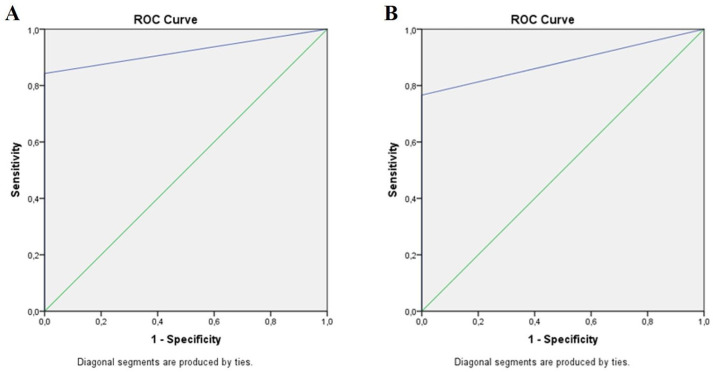
ROC curves of DR screening by photography with a hand-held fundus camera vs. standard clinical examination (**A**) and standard fundus camera (**B**). Blue line: ROC curve; green line: reference line.

In contrast to this useful clinical division of examined eyes into three groups, in the statistical calculation of all diagnostic accuracy measures, the examined eyes were divided into two grades: those with no retinopathy and those with any retinopathy (mild/moderate/severe NPDR/PDR). The shape of the ROC curves and the area under the curves (AUC) of 0.921 and 0.883 defined the excellent discriminative power of the hand-held fundus camera in DR detection compared to the standard clinical examination and very good discriminatory power compared to the standard fundus camera (Table 3, Figure 7).

Other diagnostic accuracy measures of DR detection by the hand-held camera compared to the standard clinical examination and standard fundus camera are shown in Table 4.

Photography with a hand-held camera had 89.1% and 83.2% sensitivity and 100% specificity in detecting DR compared to the clinical examination and standard camera. A positive predictive value (PPV) of 100% and a positive likelihood ratio (LR+) of infinity indicated absolute agreement between the hand-held camera and the other two methods in positively detecting patients with DR. In comparison, a negative predictive value (NPV) of 91.4% and 87.3% and negative likelihood ratio (LR−) of 0.11 and 0.17 implied almost perfect agreement between the hand-held camera and clinical examination and substantial agreement between the hand-held and standard camera in negatively noticing patients without DR. A kappa (ĸ) values of 0.86 and 0.78 and diagnostic odds ratio (DOR) values of 936.48 and 574.6 indicated almost perfect and significant agreement among the hand-held camera and clinical examination and the standard camera in detecting DR, presuming the excellent test performance of the hand-held camera. Finally, the diagnostic effectiveness (DE) of 94.9% compared to the clinical examination and 92.2% to the standard camera expressed the almost perfect accuracy of the hand-held camera in correctly classifying subjects (TP + TN) among all subjects.

## 4. Discussion

Generally, for each clinician, as well as for countries and their health systems, when choosing a method for diagnosing a disease, the diagnostic characteristics and costs of that method are fundamental. Diagnostic characteristics speak about the method’s success in correctly discriminating between certain two conditions of interest (health and disease or two stages of a disease, etc.).

In this study, the main interest was to investigate and compare the diagnostic characteristics of the small hand-held camera and AI or teleophthalmology-based grading system with a standard clinical examination and standard fundus camera in detecting DR and discriminating those persons who need the referral. This applies especially to patients with severe NPDR and PDR requiring prompt further retinal diagnostics and treatments. However, in the present study, the number (percentage) of eyes with severe NPDR and PDR detected by the hand-held camera corresponded with absolute accuracy to those detected by the clinical examination and standard camera. There was also no discrepancy in detecting eyes with moderate NPDR, whereas the substantial distinction in DR degree assessment among methods was found in the eyes with no retinopathy and those with signs of mild NPDR (single microaneurysms). Out of the 320 eyes included in the study, the hand-held camera misdiagnosed the signs of mild NPDR in 22 (6.9%) eyes screened by the standard clinical examination and in 34 (10.6%) eyes by the standard camera. Still, in both conditions, with no DR and mild NPDR, the recommendation regarding the current diabetic retinopathy guidelines is the same: regular follow-up [6,7]. Furthermore, according to all diagnostic accuracy measures used in the present study, the hand-held camera demonstrated excellent to very good discriminative power in positively detecting patients with DR and negatively detecting those without it compared to the other two diagnostic methods. Compared to the standard clinical examination, the hand-held camera had an AUC of 0.921, a sensitivity of 89.1%, and a specificity of 100%, while compared to the standard fundus camera; these measures, except for a specificity of 100%, were slightly lower: the AUC was 0.883 and the sensitivity was 83.2%.

The diagnostic accuracy of DR screening using different mobile cameras or smartphones has been widely researched, and numerous authors from various institutions and countries have obtained similar results. One of the first of such studies, published in 2015 by Rajalakshmi R et al., compared DR detection by a smartphone with the seven standard field images taken by a digital fundus camera, and found a sensitivity of 92.7% and a specificity of 98.4% for detecting any DR degree by a smartphone, while for detecting the sight-threatening diabetic retinopathy (STDR) by smartphone, the sensitivity and specificity were 87.9% and 94.9% [27]. The same authors reported that 21.9% of the images taken by a smartphone were of good quality, and the remaining 66.8% were of medium-good quality, while 61.1% of the images taken by a standard fundus camera were of good quality, and 34.9% were of medium-good quality [27]. Zhang W et al. compared screening for DR using a portable, noncontact, nonmydriatic hand-held retinal camera and the images graded by five masked ophthalmologists to the dilated clinical exam, and observed that the overall sensitivity for identifying VTDR was 64–88% and the specificity was 71–90%, while the images were gradable in 86–94% of predilation and 94–97% of postdilation photos [28]. A scoping review and meta-analysis of nine diagnostic test accuracy studies published from January 2000 to November 2018 regarding the use of various smartphone ophthalmoscopy for detecting DR reported the pooled sensitivity and specificity for detecting any DR of 87% (95% CI 74–94%) and 94% (95% CI 81–98%) and referral-warranted DR of 91% (95% CI 86–94%) and 89% (95% CI 56–98%), while the AUC ranged from 0.879 to 0.979 and the DOR from 11.3 to 1225 [29]. In the present study, the DOR for detecting DR by a hand-held camera compared to the clinical examination was 936.48, and compared to the standard camera, it was 574.6. A comparative pilot study performed in rural and tribal India, another developing country, compared the images taken with a hand-held non-mydriatic fundus camera (Horus scope 200, Medical Imaging Innovation Solution Partner, Taiwan) by two different trained technicians and graded by a retinal specialist to the indirect ophthalmoscopy completed by expert ophthalmologists and found a sensitivity, specificity, PPV, and NPV of 54.8%, 92.1%, 59.6%, and 83.9%, while the kappa agreement for DR between both detection methods was 0.48 [30]. In the present study, PPV and LR+ were 100% and infinity when comparing the hand-held camera with both diagnostic methods, while NPV and LR- were 91.4% and 0.11 when comparing the hand-held camera with the clinical examination and 87.3% and 0.17 with the standard camera. The present study’s kappa (ĸ) agreement for the comparison of the hand-held camera to clinical examination was 0.86 and 0.78 to the standard fundus camera.

In the Republic of Croatia, a country located in Central and Southeast Europe on the Adriatic Sea coast, with 327.785 adults having diabetes (8.5% of the population) [31], screening for DR is usually performed using dilated slit-lamp fundus examination only by ophthalmologists, mostly medical retina specialists, and fundus photography with a standard fundus camera is not used routinely for DR screening but only for fluorescein angiography and research purposes [32]. Also, no nurses/technicians, optometrists, new technologies such as small hand-held fundus cameras, electronic data transfer systems (including telemedicine), or AI and automated grading are introduced into DR screening in Croatia.

The hand-held fundus camera used in this study is the only one of its kind in Croatia, obtained thanks to Professor Rahelić and the IDF for the IDF Diabetic Retinopathy Screening Project which aimed to highlight the importance of integrating DR screening into routine care for people living with diabetes in developing countries. Furthermore, besides the first use of a hand-held fundus camera, this was the first time an ophthalmology nurse actively participated in DR screening in Croatia.

According to the current guidelines, screening for DR has become an important cost-effective part of diabetes care, which means that even if enough ophthalmologists are available, using ophthalmologists to screen every person with diabetes is usually not feasible and is likely to be an inefficient use of resources [6,7].

The present study determined that all three DR screening methods were simple, but the differences were in the duration of the procedure and the persons who performed them. The first author, M.T., an ophthalmologist—medical retina specialist—performed the standard clinical examination, which took the longest time, about 15–20 min for each patient. M.T. also conducted fundus photography with a standard 45° fundus camera, while Đ.H., an ophthalmology nurse, performed fundus photography with a hand-held fundus camera. Both photographing sessions lasted much shorter than the clinical examination, about 5 min each. All images (4 images of 160 subjects = 640 images in total) from the standard 45° fundus camera were subsequently and independently graded by M.T. and R.V. in about 6 h (360 min), while the images (4 images of 160 subjects = 640 images in total) from the hand-held fundus camera were graded immediately by AI and after a few hours by an IDF independent ophthalmologist.

However, besides the DR degree report, AI and an IDF ophthalmologist also evaluated the image quality and rated 77.5% of them as good quality and 22.5% as medium quality. Not a single image taken by the hand-held camera was considered low-quality or unreadable. Yet, all images (100%) taken by the standard 45° fundus camera VISUCAM Zeiss were of good quality because that nonmydriatic fundus camera has been in the Vuk Vrhovac University Clinic for the last 15 years, and the images were taken by M.T., who is very experienced in working with it. In contrast, the hand-held fundus camera TANG was entirely new at the Vuk Vrhovac University Clinic, and the photographing was performed by the ophthalmology nurse Đ.H., who was completely inexperienced in photographing with fundus cameras because in Croatia, only ophthalmologists, medical retina specialists, perform fundus photography.

All these study results implied that DR’s hand-held fundus camera assessment completed by a nurse and graded by AI or an external ophthalmologist was reliable and in agreement with those of the standard clinical examination and fundus photography with a standard camera performed by a medical retina specialist. With nurses’ further education and active participation in the photography technique and improving the image quality, it could be expected that the images would be of better quality and that the method would be more accurate and reliable.

The crucial role of nurses/technicians in DR screening with the aim of reducing the prevalence of blindness due to diabetes and reducing diabetes-related costs has been confirmed and reported earlier in numerous scientific articles and reviews [33,34,35,36]. In many diabetes centers in Europe and worldwide, educated nurses/technicians especially, in addition to fundus photographing, grade the fundus images and refer each patient with suspicious and positive results to ophthalmologists for further diagnosis and treatment. During the DR screening, the nurses/technicians also continuously educate the patients about the importance of regular DR screening and maintain good control of risk factors, such as glycemia, hypertension, and dyslipidemia, to prevent vision loss and blindness due to diabetes.

Some limitations of the present study should be addressed. First, this study was cross-sectional, performed in a single health center, and included a small number of patients. Unfortunately, all of the above were consequences of the COVID-19 pandemic onset in December 2019, when authorities worldwide recommended implementing lockdown measures to preserve public health and control the transmission of the virus. With the COVID-19 outbreak, including more participants was impossible, and the IDF Diabetic Retinopathy Screening Project ended. Second, the difference in the fundus photography experience and skill between the first author, ophthalmologist M.T., and the nurse Đ.H. resulted in a higher percentage of medium-quality images taken by the hand-held camera. Third, comparing two standard diagnostic methods, clinical examination and photography using the standard fundus camera, with a new method using an entirely new camera during only three months resulted in a deviation from the expected agreement and lower accuracy of the tested method. Fourth, we did not incorporate different imaging modalities or sources to validate the method’s versatility and explore the algorithm’s resilience to potential adversarial attacks or noisy input data. Accordingly, a nuanced understanding of each element’s contribution to the overall performance, aiding in identifying crucial components and possible areas, is missing.

## 5. Conclusions

Recently, several AI-based automated systems for DR identification have been developed and introduced. The results suggest that AI-based systems are more sensitive than human graders and could be safe to use in clinical practice. In the present study, the number (percentage) of eyes with severe NPDR and PDR detected by the hand-held fundus camera and AI-based grading system corresponded with absolute accuracy to those detected by the clinical examination and standard camera. There was also no discrepancy in detecting eyes with moderate NPDR, whereas the substantial distinction in DR degree assessment among methods was found in the eyes with no DR and those with signs of mild NPDR (single microaneurysms). In addition, the hand-held camera misdiagnosed the signs of mild NPDR in 22 (6.9%) eyes screened by the standard clinical examination and in 34 (10.6%) eyes by the standard camera. Compared to the standard clinical examination, the hand-held camera had an AUC of 0.921, a sensitivity of 89.1%, and a specificity of 100%. Implementing the DR screening using the small fundus cameras and active involvement of nurses/technicians in the DR screening process would enable all people with diabetes to be regularly screened for DR, and provide ophthalmologists—medical retina specialists—the proper time needed for further diagnostic procedures and treatments.

## Figures and Tables

**Table 1:** The basic characteristics of 320 examined eyes of 160 T2DM divided into three groups according to the level of diabetic retinopathy.

	No DR	Mild/Moderate NPDR	Severe NPDR/PDR	*p*-Value
Gender (m/f)	50/50	73/27	80/20	0.058 ^a^
Age (yrs.)	65 (48–83)	64(54–77)	62 (45–70)	0.408 ^b^
Diabetes duration (yrs.)	12.5 (2–33)	14 (7–24)	15 (2–30)	0.161 ^b^
BCVA (decimal)	0.98 ± 0.05	0.98 ± 0.08	0.89 ± 0.27	0.027 ^c^
IOP (mmHg)	14.92 ± 2.29	15.27 ± 1.16	15.20 ± 0.68	0.776 ^c^
Glaucoma (no/yes)	94/6	100/0	93/7	0.612 ^a^
Lens (1/2/3)	16/68/16	7/80/13	0/87/13	0.447 ^a^

Legend: Values are percentages, medians (min-max), or means ± SD. ^a^ indicates Chi-square test, ^b^ Kruskal–Wallis test, ^c^ one-way ANOVA test; BCVA best-corrected visual acuity; IOP intraocular pressure; Lens 1—eyes with clear crystalline lens, 2—eyes with initial cataract, 3—pseudophakic eyes.

**Table 2:** Diabetic retinopathy degree assessed by fundus photography with a hand-held fundus camera vs. standard clinical examination and photography with a standard fundus camera.

		Fundus Photography with a Hand-Held Fundus Camera		
		No Retinopathy	Mild/Moderate NPDR	Severe NPDR/PDR
Standardclinical examination	No retinopathy	180 (56.2)	0 (0)	0 (0)
	Mild/moderate NPDR	22 (6.9)	58 (18.1)	0 (0)
	Severe NPDR/PDR	0 (0)	0 (0)	60 (18.8)
Standard fundus camera	No retinopathy	168 (52.5)	0 (0)	0 (0)
	Mild/moderate NPDR	34 (10.6)	58 (18.1)	0 (0)
	Severe NPDR/PDR	0 (0)	0 (0)	60 (18.8)

N (%). Abbreviations: NPDR, nonproliferative diabetic retinopathy; PDR, proliferative diabetic retinopathy.

**Table 3:** The area under the curve for diabetic retinopathy screening by fundus photography with a hand-held fundus camera vs. standard clinical examination and standard fundus camera.

	Fundus Photography with a Hand-Held Fundus Camera			
	Area	Std. Error	95% CI	*p*
Standard clinical examination	0.921	0.026	0.870–0.973	0.000
Standard fundus camera	0.883	0.030	0.824–0.942	0.000

**Table 4:** Diagnostic accuracy measures of diabetic retinopathy detection by the hand-held fundus camera vs. standard clinical examination and standard fundus camera.

Hand-Held Fundus Camera vs.	Standard Examination		Standard Fundus Camera	
	Estimate	95% CI	Estimate	95% CI
Sensitivity	89.1%	81.3–94.4%	83.2%	74.4–89.9%
Specificity	100%	93.9–100%	100%	93.9–100%
PPV	100%	95.9–100%	100%	95.7–100%
NPV	91.4%	85.9–94.9%	87.3%	81.7–91.4%
LR+	Infinity	NaN-Infinity	Infinity	NaN-Infinity
LR−	0.11	0.06–0.19	0.17	0.11–0.26
Kappa ± SE	0.86 ± 0.04	0.77–0.94	0.78 ± 0.05	0.69–0.88
DOR	936.48	54.2–16194.6	574.6	33.8–9743.5
DE	94.9%	90.3–97.8%	92.2%	86.9–95.8%

Abbreviations: PPV—positive predictive value; NPV—negative predictive value; LR+—positive likelihood ratio; LR−—negative likelihood ratio; Kappa—kappa agreement; DOR: diagnostic odds ratio; DE—diagnostic effectiveness (accuracy); NaN—the calculation cannot be performed because the values entered include one or more instances of zero.

## Data Availability

The data presented in this study are available upon specific request from the corresponding author.

## References

[1] International Diabetes Federation (2021). IDF Diabetes Atlas.

[2] Flaxman S.R., Bourne R.R.A., Resnikoff S., Ackland P., Braithwaite T., Cicinelli M.V., Das A., Jonas J.B., Keeffe J., Kempen J.H. (2017). Global causes of blindness and distance vision impairment 1990–2020: A systematic review and meta-analysis. Lancet Glob. Health.

[3] Teo Z.L., Tham Y.C., Yu M., Chee M.L., Rim T.H., Cheung N., Bikbov M.M., Wang Y.X., Tang Y., Lu Y. (2021). Global prevalence of diabetic retinopathy and projection of burden through 2045: Systematic review and meta-analysis. Ophthalmology.

[4] Javitt J.C., Aiello L.P. (1996). Cost-effectiveness of detecting and treating diabetic retinopathy. Ann. Intern. Med..

[5] Rohan T.E., Frost C.D., Wald N.J. (1989). Prevention of blindness by screening for diabetic retinopathy: A quantitative assessment. BMJ.

[6] Wong T.Y., Sun J., Kawasaki R., Ruamviboonsuk P., Gupta N., Lansingh V.C., Maia M., Mathenge W., Moreker S., Muqit M.M.K. (2018). Guidelines on diabetic eye care: The international council of ophthalmology recommendations for screening, follow-up, referral, and treatment based on resource settings. Ophthalmology.

[7] World Health Organization (2020). Regional Office for Europe (WHO/Europe). Diabetic Retinopathy Screening: A Short Guide: Increase Effectiveness, Maximize Benefits and Minimize Harm.

[8] Garg S., Jani P.D., Kshirsagar A.V., King B., Chaum E. (2012). Telemedicine and retinal imaging for improving diabetic retinopathy evaluation. Arch. Intern. Med..

[9] Shi L., Wu H., Dong J., Jiang K., Lu X., Shi J. (2015). Telemedicine for detecting diabetic retinopathy: A systematic review and meta-analysis. Br. J. Ophthalmol..

[10] Jin K., Lu H., Su Z., Cheng C., Ye J., Qian D. (2017). Telemedicine screening of retinal diseases with a handheld portable non-mydriatic fundus camera. BMC. Ophthalmol..

[11] Padhy S.K., Takkar B., Chawla R., Kumar A. (2019). Artificial intelligence in diabetic retinopathy: A natural step to the future. Indian J. Ophthalmol..

[12] US Food and Drug Administration (2018). FDA Permits Marketing of Artificial Intelligence-Based Device to Detect Certain Diabetes-Related Eye Problems.

[13] Bhaskaranand M., Ramachandra C., Bhat S., Cuadros J., Nittala M.G., Sadda S.R., Solanki K. (2019). The value of automated diabetic retinopathy screening with the EyeArt system: A study of more than 100,000 consecutive encounters from people with diabetes. Diabetes Technol. Ther..

[14] Wang X.N., Dai L., Li S.T., Kong H.Y., Sheng B., Wu Q. (2020). Automatic grading system for diabetic retinopathy diagnosis using deep learning artificial intelligence software. Curr. Eye Res..

[15] Wang Y., Shi D., Tan Z., Niu Y., Jiang Y., Xiong R., Peng G., He M. (2021). Screening referable diabetic retinopathy using a semi-automated deep learning algorithm assisted approach. Front. Med..

[16] Orujov F., Maskeliūnas R., Damaševičius R., Wei W. (2020). Fuzzy based image edge detection algorithm for blood vessel detection in retinal images. Appl. Soft Comput..

[17] Hu K., Zhang Z., Niu X., Zhang Y., Cao C., Xiao F., Gao X. (2018). Retinal vessel segmentation of color fundus images using multiscale convolutional neural network with an improved cross-entropy loss function. Neurocomputing.

[18] Jiang Z., Yepez J., An S., Ko S. (2017). Fast, accurate and robust retinal vessel segmentation system. Biocybern. Biomed. Eng..

[19] Liu H., Wei D., Lu D., Li Y., Ma K., Wang L., Zheng Y. (2021). Simultaneous Alignment and Surface Regression Using Hybrid 2D-3D Networks for 3D Coherent Layer Segmentation of Retina OCT Images. Medical Image Computing and Computer Assisted Intervention—MICCA 2021I.

[20] Apon T.S., Hasan M.M., Islam A., Alam G.R. Demystifying Deep Learning Models for Retinal OCT Disease Classification using Explainable AI. Proceedings of the 2021 IEEE Asia-Pacific Conference on Computer Science and Data Engineering (CSDE).

[21] Zhelev Z., Peters J., Rogers M., Allen M., Kijauskaite G., Seedat F., Wilkinson E., Hyde C. (2023). Test accuracy of artificial intelligence-based grading of fundus images in diabetic retinopathy screening: A systematic review. J. Med. Screen..

[22] The Fred Hollows Foundation, International Diabetes Federation (2015). Diabetes Eye Health: A Guide for Health Professionals.

[23] Scanlon P.H., Malhotra R., Greenwood R.H., Aldington S.J., Foy C., Flatman M., Downes S. (2003). Comparison of two reference standards in validating two field mydriatic digital photography as a method of screening for diabetic retinopathy. Br. J. Ophthalmol..

[24] Boucher M.C., Gresset J.A., Angioi K., Olivier S. (2003). Effectiveness and safety of screening for diabetic retinopathy with two nonmydriatic digital images compared with the seven standard stereoscopic photographic fields. Can. J. Ophthalmol..

[25] Wilkinson C.P., Ferris F.L., Klein R.E., Lee P.P., Agardh C.D., Davis M., Dills D., Kampik A., Pararajasegaram R., Verdaguer J.T. (2003). Proposed international clinical diabetic retinopathy and diabetic macular edema disease severity scales. Ophthalmology.

[26] Shanghai Jiao Tong University Project 27: Screening and Auxiliary Diagnosis System of Diabetic Retinopathy based on Artificial Intelligence. https://global.sjtu.edu.cn/en/studyatSJTU/session/436.

[27] Rajalakshmi R., Arulmalar S., Usha M., Prathiba V., Kareemuddin K.S., Anjana R.M., Mohan V. (2015). Validation of smartphone-based retinal photography for diabetic retinopathy screening. PLoS ONE.

[28] Zhang W., Nicholas P., Schuman S.G., Allingham M.J., Faridi A., Suthar T., Cousins S.W., Prakalapakornet S.G. (2017). Screening for diabetic retinopathy using a portable, noncontact, nonmydriatic handheld retinal camera. J. Diabetes Sci. Technol..

[29] Tan C.H., Kyaw B.M., Smith H., Tan C.S., Tudor Car L. (2020). Use of smartphones to detect diabetic retinopathy: Scoping review and meta-analysis of diagnostic test accuracy studies. J. Med. Internet Res..

[30] Gajiwala U.R., Pachchigar S., Patel D., Mistry I., Oza Y., Kundaria D., Shamanna B.R. (2022). Non-mydriatic fundus photography as an alternative to indirect ophthalmoscopy for screening of diabetic retinopathy in community settings: A comparative pilot study in rural and tribal India. BMJ Open.

[31] Poljičanin T., Buble T. (2022). National Diabetes Registry CroDiab—Report for 2021.

[32] Tomić M., Raštegorac P., Vrabec R., Poljičanin T., Rahelić D. (2020). Telemedicine for diabetic retinopathy screening in Croatia: A dream that could become a reality. Coll. Antropol..

[33] Connolly A., Hosker C. (2001). The nurse’s role in screening for diabetic retinopathy. Nurs. Times.

[34] Kirkwood B.J., Coster D.J., Essex R.W. (2006). Ophthalmic nurse practitioner led diabetic retinopathy screening. Results of a 3-month trial. Eye.

[35] Spurr S., Bullin C., Bally J., Trinder K., Khan S. (2018). Nurse-led diabetic retinopathy screening: A pilot study to evaluate a new approach to vision care for Canadian Aboriginal peoples. Int. J. Circumpolar. Health.

[36] Boucher M.C., Nguyen M.T.D., Qian J. (2020). Assessment of training outcomes of nurse readers for diabetic retinopathy telescreening: Validation study. JMIR Diabetes.

